# Diversity of Fungal Communities in Heshang Cave of Central China Revealed by Mycobiome-Sequencing

**DOI:** 10.3389/fmicb.2018.01400

**Published:** 2018-07-16

**Authors:** Baiying Man, Hongmei Wang, Yuan Yun, Xing Xiang, Ruicheng Wang, Yong Duan, Xiaoyu Cheng

**Affiliations:** ^1^State Key Laboratory of Biogeology and Environmental Geology, China University of Geosciences, Wuhan, China; ^2^College of Life Science, Shangrao Normal University, Shangrao, China; ^3^Laboratory of Basin Hydrology and Wetland Eco-restoration, China University of Geosciences, Wuhan, China

**Keywords:** subterranean karst ecosystem, geomicrobiology, ITS1 region amplicons, mycobiomes, Illumina HiSeq sequencing, Heshang Cave

## Abstract

Deciphering of the mycobiome in pristine karst caves has been impeded by constraints of remote locations, inaccessibility to specimens and technical limitations, which greatly restricted in-depth understanding of mycobiomes in subterranean ecosystem. Here, mycobiomes of Heshang Cave in south-western karst region of China were investigated by Illumina HiSeq sequencing of fungal rRNA-ITS1 gene across different habitats. In total 793,502 ITS1 reads and 2,179 OTUs from 778 Mb reads after stringent quality control (Q30) and 453 genera, 72 orders and 19 classes within 6 phyla were detected. Ascomycota (42% OTUs) dominated across the five habitats. Shannon-Wiener index varied from 1.25 to 7.62 and community richness was highest in drip waters, followed by weathered rocks, bat guanos, sediments, and air samples. Mycobiomes displayed specificity to five habitats and more distinct OTUs were found in weathered rocks (12%) and drip waters (9%). In contrast, only 6.60% core OTUs were shared by five habitats. Notably, weathered rocks possessed more indicator groups and were revealed for the first time to be dominated by Sordariomycetes (43%). The community richness of air mycobiomes increased from cave entrance to the innermost part and dominated by the indicator groups of *Penicillium mallochii* (>30%) and *P. herquei* (>9%). Our work represents the largest attempt to date to a systematical investigation of oligotrophic solution-cave-associated mycobiomes in China. Our discovery of high diversity of mycobiomes in Heshang Cave also suggests that eukaryotic microorganisms may play a crucial role in subsurface environments.

## Introduction

As the second largest kingdom of eukaryotic life, fungi are more diverse and widely distributed in aquatic and terrestrial ecosystems, such as soils (Ritz and Young, 2004), lakes and deep-sea (Shearer et al., 2007; Nagahama and Nagano, 2012), caves and rock surfaces (Sterflinger, 2000; Gorbushina, 2007; Vanderwolf et al., 2013). Mycobiomes at global-scale indicated a regional endemism patterns (Meiser et al., 2014) and strongly influenced by bio-geographical factors such as climates (Tedersoo et al., 2014) and geographical isolation (Talbot et al., 2014). Although exploration of the mycobiome has been greatly facilitated by the development of the methodology, only a small portion has been discovered in comparison to the estimated 1.5–5.1 million species (Hawksworth, 2001). Even if great efforts have been put on uncovering the diversity of mycobiomes in different niches via next generation sequencing (NGS) technologies (Lentendu et al., 2011; Shi et al., 2014; Duarte et al., 2015; Abdelfattah et al., 2016), it still lags behind in comparison with those of the prokaryotic microbiomes. Increasing investigations via NGS techniques in recent years successfully revealed the diversity of the prokaryotic microbiomes (Zhang et al., 2012; Blasiak et al., 2014; Walsh et al., 2016; Yun et al., 2016a) and provided enormous amount of data on the premise of low price (Caporaso et al., 2012; Degnan and Ochman, 2012), while few NGS studies focused on systematic investigations of the diversity of mycobiomes in pristine karst caves (Vaughan et al., 2015). Fungi have long been recorded in caves 225 years ago (Dobat, 1967), however, they have received far less attention and limited our deeper understanding for the diversity and potential roles of fungi in subterranean ecosystems.

Karst terrain accounts for a fifth of the world’s dry, ice-free land, and south-western China hosts the largest contiguous karst areas in the world with a karst area of 5.0 × 10^5^ km^2^ (Derek and Williams, 2007). Pristine karst caves are generally considered as extreme subterranean environments for the development of life (Lavoie and Northup, 2001) due to the geographical isolation, the absence of light and low organic carbon available for metabolic activities (Gherman et al., 2014). For cave mycological studies, only 1,029 species in 518 genera of fungi, slime molds and fungus-like taxa have been reported to date in solution caves (Vanderwolf et al., 2013). Most importantly, 91.5% fungal ecological studies of solution caves were primarily based on culture-dependent methods (Vanderwolf et al., 2013) and more than 99% of microbial taxa have yet to be discovered (Locey and Lennon, 2016). This substantially limits in-depth knowledge of solution cave mycobiomes *in situ* as well as further exploration of fungal potential ecological functions.

Heshang Cave locates in the middle reaches of the Yangtze River in south-western karst region of China. It is characterized by slight alkalinity (pH 8.2–8.7), darkness and constant temperature (16–18°C). Since 1997, significant progresses have been made on understanding of bacterial communities (Liu et al., 2010; Gong et al., 2015; Yun et al., 2016b) and their interaction with minerals in Heshang Cave (Wang et al., 2010; Zhou et al., 2017). In contrast, mycobiomes were poorly characterized and 10 unique fungal genera were revealed in Heshang Cave (Man et al., 2015).

Sequencing data of mycobiomes quickly increased during the past decade and the internal transcribed spacer (ITS) region of nuclear DNA was usually used as the phylogenetic marker for fungi (Schoch et al., 2012). Particularly, the ITS1 sub-region was employed by the microbiome projects (Tedersoo and Lindahl, 2016) and successfully recovered mycobiomes in different niches (Metcalf et al., 2016; Walters et al., 2016). To date, systematic investigation of cave-associated mycobiomes using Illumina HiSeq sequencing techniques has been rarely documented which limits our understanding about the fungal diversity and their potential ecological functions in the unique cave ecosystems. Therefore, the specific goals of this work was to (1) in-depth explore the hidden mycobiomes of the air, weathered rocks, bat guanos, sediments, and drip waters in Heshang Cave using fungal barcode marker ITS1 coupled with Illumina HiSeq PE250 sequencing technology and (2) reveal the diversity of mycobiomes and community differences in five habitats of Heshang Cave and address a new insight into fungal communities under nutrient-limited solution cave ecosystems.

## Materials and Methods

### Study Sites and Sample Collection

**FIGURE 1:**
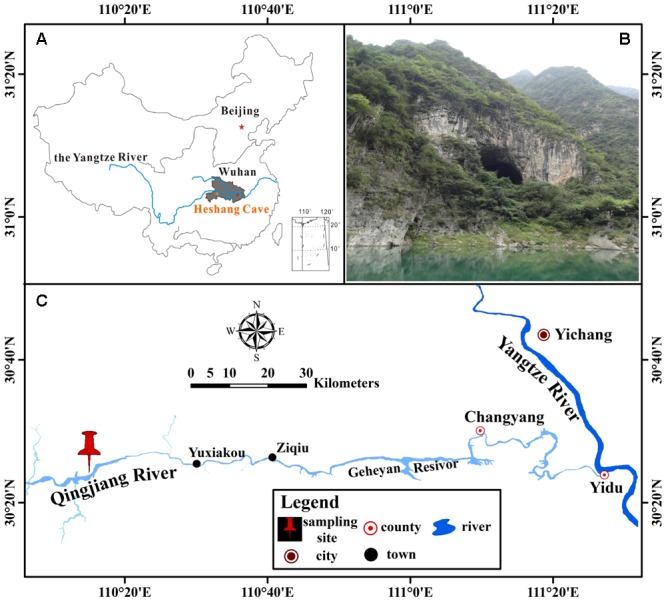
Location of Heshang Cave, central China. **(A)** The dark gray area shows the location of Hubei province. **(B)** Outside view of Heshang Cave. **(C)** Location of Heshang Cave.

Heshang Cave (30°27′ N, 110°25′ E), a pristine carbonate cave, locates in the south bank of Qingjiang Valley in the middle reaches of the Yangtze River, south-western karst region, China (**Figures 1A,C**). It developed in Cambrian dolomite, horizontally oriented and overlain by ∼400 m of Cambrian dolomite. The cave is about 250 m long, with a sole natural entrance (∼20 m in width and height) and only accessible by boat from the Qingjiang River (**Figure 1B**). It is a slight alkaline, dark, and oligotrophic cave with an intermittent subterranean stream and active drip waters, which is affected by East Asian monsoon in this karst region. The inner annual mean temperature in Heshang Cave remains between 16 and 18°C (Hu et al., 2008).

**FIGURE 2:**
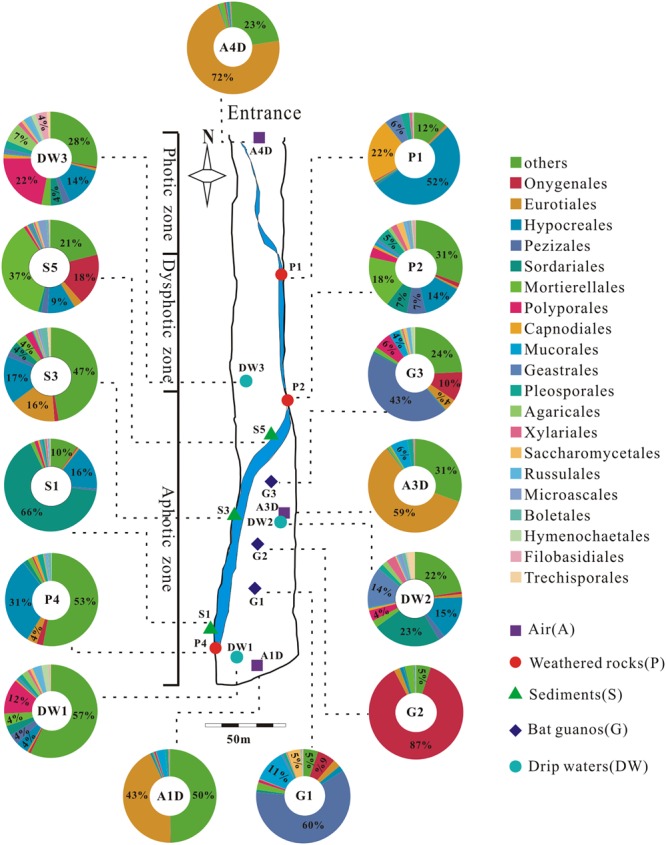
Schematic map of sampling sites and the relative abundance (≥4%) pie chart of the top 20 known fungal orders detected in 15 samples in Heshang Cave. Others in each pie chart indicate the sum of the relative abundance of the rest orders. Abbreviations: A: air; P: weathered rocks; S: sediments; G: bat guanos; DW: drip waters.

A total of 39 samples were collected at 15 sampling sites across five habitats, including sediments, weathered rocks, bat guanos, drip waters, and air in Heshang Cave (**Figure 2**). Triplicate samples were collected aseptically at each site by five-point sampling method for the solid samples. Only three samples of drip waters were collected due to lack the active dripping sites (based on direct observation) during sampling time. Briefly, samples of sediments (S1/S3/S5), bat guanos (G1/G2/G3) (in aphotic zone), and weathered rocks (P1/P2/P4) (P1: twilight zone; P2/P4: aphotic zone) (**Figure 2**) were collected aseptically with 50 ml sterile plastic centrifuge tubes (Corning). Samples of drip waters (DW1/DW2/DW3) were collected with 10-L sterile plastic bottles in the aphotic zone (DW1/DW2) and twilight zone (DW3), respectively (**Figure 2**). We filtered the drip waters with sterilized membranes (0.22 μm) within 48 h and stored the membranes in 50 ml sterile plastic centrifuge tubes at -80°C. For air sampling, a six-stage BY-300 sampler [aerodynamic diameter (*d_a_*) = 0.65–1.1, 1.1–2.1, 2.1–3.3, 3.3–4.7, 4.7–7.0, and > 7.0 μm; Pusen electronic machine factory, Changzhou, China] with air flow rate of 28.3 L min^-1^ was used to collect airborne fungi. The sampler was calibrated before use and mounted 1.5 m above ground level before sampling. Aerosol samples (A1D/A3D: aphotic zone; A4D: photic zone) were collected on sterilized membranes in six-stage of sampler for 5 min, three times at each sampling site (**Figure 2**). The membranes were kept in 50 ml sterile plastic centrifuge tubes after sampling and transported to the geomicrobiology laboratory in China University of Geosciences (Wuhan) under the refrigeration within 48 h and stored at -80°C for DNA extraction.

### Physicochemical Parameters

The pH of drip waters was measured *in situ* with a multiparameter water quality detector (HACH, Loveland, CO, United States). Temperature, humidity, and the concentration of carbon dioxide for cave air from entrance to the innermost were detected in field. pH of solid samples was measured with a UB-7 pH meter (Denver Instrument) in the laboratory. The concentrations of dissolved major anions and cations were analyzed using an ICS-900 ion chromatograph (Thermo Fisher, USA). The mineral phases of weathered rocks and sediments were analyzed by X-ray diffraction (XRD, Bruker AXS D8-Focus, Germany).

### Genomic DNA Extraction

We used 0.5 g subsamples of sediments, weathered rocks, and bat guanos for genomic DNA extraction with the FastDNA^®^ SPIN Kit for Soil (MP Bio, Santa Ana, CA, United States) after the samples were freeze-dried (ALPHA 1-2 LD, Christ, Lower Saxony, Germany). For samples of air and drip waters, the membranes with the filtered cells were used for DNA extraction by the PowerWater^®^ DNA Isolation Kit (Mobio Laboratory, Carlsbad, CA, United States). Extracted nucleic acids were eluted with 60 μl buffer and quantified with Nanodrop (Thermo Scientific, United States). The isolated DNA of final concentration of 1 ng/μl was used as templates for subsequent polymerase chain reactions (PCRs). Negative controls were processed in parallel with all samples to avoid contamination from reagents and background signals. Results shown here are only from those samples, which passed PCR-test and controls.

### Amplicon Preparation and Illumina HiSeq Sequencing

We designed six-nucleotide-long error-correcting barcode (denoted by x) appended 5′-ends of universal fungal primer pairs ITS5-1737F (5′-xxxxxxGGAAGTAAAAGTCGTAACAAGG-3′)/ITS2-2043R (5′-xxxxxxGCTGCGTTCTTCATCGATGC-3′) (Bellemain et al., 2010), which target the phylogenetic marker ITS1 (Nilsson et al., 2009). Briefly, ITS5-1737F was selected as the forward primer due to its high recovery efficiency in comparison with ITS1-F (Bellemain et al., 2010) and the absence of the ITS1 intron (Bazzicalupo et al., 2013). The 5′ end of forward fusion primer was incorporated with a 6-bp tag sequence, while the 5′ end of reverse primers was marked with a 6-bp sample-identifying barcode sequences unique to each sample (Supplementary Table 1). We designed 15 different fusion primers in total for this experiment. Diluted (1 ng/μl) genomic DNA samples were amplified separately using the fusion primer pairs and Phusion^®^ High-Fidelity PCR Master Mix (New England Biolabs) under the PCR conditions described previously (Man et al., 2015). PCR products of triplicate reactions for each sample were visualized on 2% agarose gels and pooled in equimolar ratios, then purified with Qiagen Gel Extraction Kit (Qiagen, Germany). Separate extractions, PCRs, and pooling for individual subsamples were conducted to reduce PCR bias. A total of 15 pooled samples were obtained after mixed the PCR products of each subsamples. Paired-end libraries of pooled samples were generated using TruSeq^®^ DNA PCR-Free Sample Preparation Kit (Illumina, United States) following manufacturer’s recommendations and Illumina sequencing adaptors were added. No sequence was retrieved for controls in the paired end libraries. The barcode in the PCR primer was marked and adaptors were added to the PCR products by ligation to reduce the length of primers to <30 bp, and ensure the standard PCR programs working. The library quality was assessed on the Qubit@ 2.0 Fluorometer (Thermo Scientific) and Agilent Bioanalyzer 2100 system. Sequencing of 15 representative ITS1 tag-encoded amplicon pools were then performed commercially on Illumina HiSeq 2500 platform at the Novogene Bioinformatics Technology (Beijing, China) and 250 bp paired-end reads were generated.

### Bioinformatics Analysis

Fastq files (forward: fastq1, reverse: fastq2) of raw reads for 15 pooled samples were provided by the paired-end Illumina HiSeq 2500 run. After demultiplexed and truncated the barcode and primer sequence, reads were merged using FLASH^1^ (Version 1.2.7) (Magoč and Salzberg, 2011) to generate splicing sequences (raw tags). We applied a comparatively stringent quality control of raw tags and removed the sequences with low Phred quality scores (< 30) and more than one ambiguous calls (N). Clean tags were obtained using QIIME^2^ (Version 1.7.0) (Caporaso et al., 2010) after quality control, and then compared with the UNITE^3^ Database using UCHIME^4^ algorithm (Edgar et al., 2011) to detect chimera sequences. All samples were normalized at the same sequence depth (30527) after chimera sequences removed, and the effective tags (Haas et al., 2011) were clustered into OTU at 97 and 95% similarity homology with UPARSE^5^ (Version 7.0.1001) (Edgar, 2013). Representative sequence for each OTU was screened for further annotation using QIIME (Version 1.7.0) based on Blast algorithm in the UNITE^3^ Database and taxonomic affiliation (Kõljalg et al., 2013). Phylogenetic trees were constructed by the MUSCLE^6^ software (Version 3.8.31) (Edgar, 2004).

The raw sequence datasets were submitted to the NCBI^7^ Sequence Read Archive (Kodama et al., 2012), accession no. SRP092436.

### Biodiversity Assessment of Mycobiomes

Sequences of OTUs with an identity of 95% were used in subsequent analyses unless otherwise declared. Genotype groups based on 95% sequence homology were used here as the conservative estimate for intraspecific divergence (U’ren et al., 2009) and avoid the diversity overestimation caused by variable ITS sequences (Man et al., 2015). Diversity indexes and richness metrics of samples grouped at 97% sequence homology were also listed to explore the congruence of diversity at two levels of cut-off (**Table 1**).

**Table 1:** The sequencing results of mycobiomes for 15 samples in Heshang Cave. Comparison of diversity indices (Shannon, Simpson) and richness metrics (Observed-species, Chao1, ACE) at 95 and 97% similarity homology are also presented.

Sample	Total tags	Taxon tags	OTUs		Observed species		Shannon		Simpson		Chao1		ACE		Good’s coverage (%)	
			3%	5%	3%	5%	3%	5%	3%	5%	3%	5%	3%	5%	3%	5%
A1D	63996	63626	367	338	262	242	3.06	2.94	0.75	0.74	337.62	329.57	352.91	341.10	99.70	99.70
A3D	59125	58944	224	198	170	161	3.11	2.76	0.81	0.75	235.56	207.58	237.42	232.51	99.80	99.80
A4D	55879	55703	242	211	210	183	3.32	3.11	0.84	0.81	307.53	257.29	310.24	261.24	99.80	99.70
Mean A	59667	59424	278	249	214	195	3.16	2.94	0.80	0.88	293.57	264.81	300.19	278.28	99.77	99.73
P1	57944	57678	653	606	530	491	4.65	4.56	0.90	0.90	718.54	648.12	704.41	666.89	99.50	99.40
P2	44103	42816	1495	1360	1335	1195	7.78	7.62	0.99	0.99	1464.27	1299.84	1535.05	1349.12	99.20	99.10
P4	31341	30527	567	516	567	491	4.97	4.81	0.88	0.86	620.16	540.76	626.54	521.29	99.80	99.70
Mean P	44463	43674	905	827	811	726	5.80	5.66	0.92	0.92	934.32	829.57	955.33	845.77	99.50	99.40
S1	63997	63706	633	584	470	446	2.81	2.78	0.56	0.56	556.29	557.69	576.88	566.67	99.60	99.60
S3	33391	33039	514	477	429	414	5.24	5.22	0.94	0.94	488.92	464.28	492.41	479.23	99.70	99.70
S5	70034	69455	975	897	697	652	5.42	5.33	0.94	0.94	924.03	911.92	937.48	879.13	99.30	99.20
Mean S	55807	55400	707	653	532	504	4.49	4.44	0.81	0.81	656.41	644.63	668.92	641.68	99.53	99.50
G1	61366	61090	1000	921	706	648	3.47	3.47	0.67	0.68	1006.99	877.36	1033.58	924.95	99.20	99.10
G2	48432	48050	358	333	285	270	1.33	1.25	0.25	0.24	388.33	355.21	389.95	368.15	99.70	99.70
G3	53309	53037	649	607	545	522	4.17	4.11	0.81	0.81	716.11	687.07	722.44	696.38	99.40	99.40
Mean G	54369	54059	669	620	512	480	2.99	2.94	0.58	0.58	703.81	639.88	715.32	663.16	99.43	99.40
DW1	48064	47455	1231	1116	1065	985	6.37	6.20	0.93	0.93	1358.93	1146.15	1354.10	1189.98	99.20	99.00
DW2	62057	61522	1120	1020	863	801	6.41	6.32	0.95	0.95	1049.60	998.06	1066.23	981.10	99.40	99.30
DW3	47238	46854	1173	1064	1022	923	7.38	7.27	0.98	0.98	1265.34	1075.88	1250.65	1080.88	99.40	99.20
Mean D	52453	51944	1175	1067	983	903	6.72	6.60	0.95	0.95	1224.62	1073.36	1223.66	1083.99	99.33	99.17
**Total**	800276	793502	11201	11248	9126	8424										
**Mean**	53352	52900	747	683	610	562	4.63	4.52	0.81	0.83	762.55	690.45	772.69	702.58	99.51	99.44

Generally, statistical analyses of alpha diversity for community richness (Observed-species, Chao1, ACE), community diversity (Shannon, Simpson) and Coverage (Good’s coverage) and beta diversity (weighted and unweighted Unifrac) were estimated using rarefied data with QIIME^2^ (Version 1.7.0). Differences in alpha diversity for all pairwise difference between means were compared by parameter (Tukey’s test) and nonparametric (Wilcoxon’s test) statistical tests. In addition, a significance test for habitat based beta diversity was performed by PERMANOVA analysis. Community composition was performed by methods of nonmetric multidimensional scaling (NMDS) and principal coordinate analysis (PCoA) in R software (Version 2.15.3). The distinctiveness of fungal groups in different habitats was statistically analyzed by linear discriminant analysis effect size (LEfSe) method (Segata et al., 2011). The taxon with significant difference between groups and significance of the detected variations were analyzed by *t*-test (*p*-value) and Metastats test (*p*-value and *q*-value).

## Results

### Physicochemical Parameters in Heshang Cave

The mean temperature of air is 18°C. The relative humidity and concentrations of CO_2_ are increasing from entrance (96%, 418 ppm) to the innermost part (100%, 494 ppm). All solid and liquid samples other than bat guanos were slightly alkaline, with pH ranging from 7.74 to 8.15. Drip waters have the lowest concentrations of Ca^2+^, Mg^2+^, Cl^-^, NO_3_^-^, and SO_4_^2-^ compared with other samples (Supplementary Table 2). Higher concentrations of Ca^2+^ and Mg^2+^, ranging from 2.34 to 11.90 mM and 2.65 to 24.25 mM, respectively, were observed in weathered rocks and bat guanos. Bat guano have the highest NO_3_^-^, ranging from 11.90 to 38.22 mM (Supplementary Table 2). Minerologically weathered rocks and sediments were dominated by dolomite with minor portion of fluorapatite and quartz, respectively.

### Assessment of Sequence Data

A total of 15 pooled samples representing five different habitats in Heshang Cave were sequenced by Illumina HiSeq 2500 platform, resulting in 778 Mb reads and 1,012,715 sequences. Of those, 800,276 (79%) high-quality chimera-free effective tags (244 bp on average) passed the stringent quality control (Phred quality scores 30: 98% on average) and fell into 793,502 taxa tags (52,900 on average) and 11,201 OTUs (747 on average) (**Table 1**). The mean effective tags retrieved from air (59,667), sediment (55,807) and bat guano (54,369) samples were higher than mean tags (53,352) of total samples, while mean effective tags of weathered rocks (44,463) and drip waters (52,453) were lower than the mean value (**Table 1**). The same trend was observed in taxon tags clustering at 95% and 97% similarity thresholds (**Table 1**). However, the number of different phylogenetic OTUs was not correlated with the number of effective tags. The mean number of OTUs was highest in drip waters (1,067/1,175), followed by weathered rocks (827/905), sediments (653/707), bat guanos (620/669) and air (249/278) at 95 and 97% similarity threshold, respectively (**Table 1**). Notably, sequences of weathered rocks only account for 17% of total effective tags (44,463 on average), but possessed much higher OTU numbers (totally 2,482 OTUs in weathered rocks and averaged at 827 OTUs) (**Table 1**). OTU numbers at 95% sequence similarity averaged at 683 of all samples analyzed, with different phylogenetic OTUs ranging from a minimum of 198 OTUs in air subsamples to a maximum of 1360 OTUs in weathered rock subsamples (**Table 1**).

### Taxonomy and Community Composition

**FIGURE 3:**
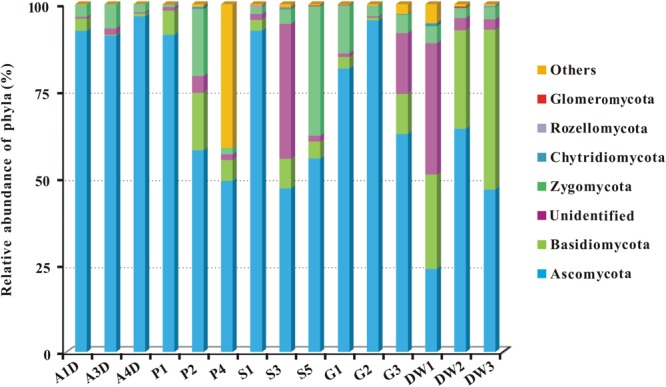
The relative abundance of mycobiomes at the phylum level in Heshang Cave. Abbreviations are the same as those in **Figure 2**.

Representative sequences of 2,179 OTUs were annotated from phylum to genus levels using the default setting of QIIME software and the main taxonomy of them were listed in Supplementary Table 3. The mean OTU numbers in 5 different habitats ranged from 249 to 1067 (**Table 1**) with 5% cutoff. In total 453 known genera, 72 known orders and 19 known classes in 6 phyla of fungi were detected in 15 samples of Heshang Cave (Supplementary Table 3). Only 6.43 and 2.35% of sequences belong to unknown fungi and no blast hits, respectively (Supplementary Table 3). Ascomycota was the most dominant phylum with a relative abundance of 72.25% (573,274 reads, 916 OTUs), followed by Basidiomycota (10.66%, 84,614 reads, 653 OTUs), Zygomycota (8.02%, 63,647 reads, 54 OTUs), Chytridiomycota (0.14%, 1,081 reads, 32 OTUs), Rozellomycota (0.09%, 706 reads, 15 OTUs), and Glomeromycota (0.06%, 498 reads, 11 OTUs). The relative abundance of detected fungal taxa at the phylum level for 15 samples was showed in **Figure 3**. Among the 19 discriminated fungal classes, 9 belonged to Ascomycota, 7 to Basidiomycota, 2 to Glomeromycota, and 1 to Chytridiomycota. Unidentified and *incertae sedis* at the class level were 17.89 and 7.71%, respectively (Supplementary Table 3).

At the order level, in total of 72 known orders were obtained in investigated habitats. Of them, 11% high-abundance orders (with >50 OTUs in each order) encompassed nearly 42 and 36% of total reads and OTUs, respectively, and the relative abundances varied from 2 to 8.50%. By contrast, 26% medium-abundance orders (10 to 50 OTUs in each order) occupied 31 and 23% of total reads and OTUs, respectively, and the relative abundances varied from 0.40 to 2%. The remaining 45 fungal taxa were rare, only accounted for 3.40 and 5.60% of total reads and OTUs, and their relative abundances varied from 0.001 to 0.20% (Supplementary Table 3).

We detected 34, 26, 5, 4, and 3 known orders in Ascomycota, Basidiomycota, Zygomycota, Chytridiomycota, and Glomeromycota, respectively. The relative abundance of identified orders in terms of their reads and OTUs was shown in Supplementary Table 3, in which Eurotiales (14.92%, 118,359 reads, 54 OTUs) was the most abundant, while Agaricales (0.95%, 7,505 reads, 120 OTUs) in Basidiomycota was the least abundant. Chytridiomycota and Glomeromycota were represented by Chytridiales (654 reads, 5 OTUs) and Glomerales (20 reads, 3 OTUs), respectively. The relative abundance of the top 20 orders in 15 samples in Heshang Cave was shown in **Figure 2**.

### Core Genera of Mycobiomes

**FIGURE 4:**
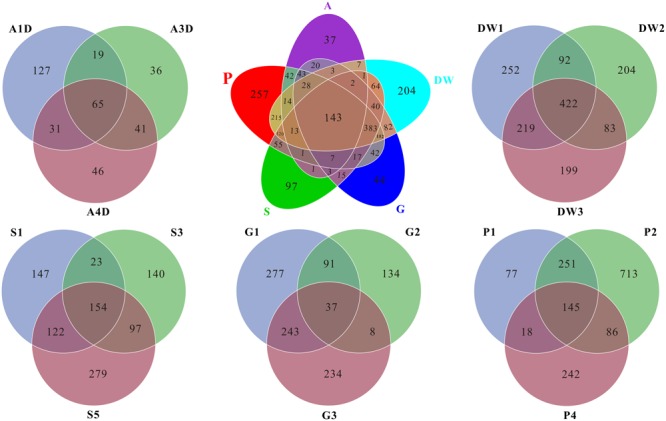
Venn diagram displayed the number of common and distinct OTUs for mycobiomes of five habitats in Heshang Cave. Abbreviations are the same as those in **Figure 2**.

As for the shared and unique core OTUs (i.e., the OTU observed in three subsamples of a given habitat) in investigated habitats, only 6.60% core OTUs were common in all samples, but more distinct OTUs were observed in specific samples, especially in weathered rocks (11.79%) and drip waters (9.36%) (**Figure 4**). These results further illustrated a high genetic diversity of fungi in Heshang Cave. The top 10 genera present in Heshang Cave were mainly distributed in 3 phyla:*Penicillium* (11.21%), *Chaetomium* (6.88%), *Neogymnomyces* (5.47%), *Aspergillus* (2.72%), *Mallochia* (1.41%), and *Gymnoascus* (1.11%) in Ascomyceta; followed by *Mortierella* (5.92%), *Mucor* (0.94%), and *Circinella* (0.86%) in Zygomycota and *Geastrum* (1.83%) in Basidiomycota.

Mycobiomes in Heshang Cave are highly diverse with 453 known genera in total. All the samples shared 6.2% common OTUs at the genus level. Weathered rocks and drip waters possessed more unique OTUs in comparison with other samples (Supplementary Figure 1). Core genera (i.e., the genus observed in three subsamples of a given habitat) varied significantly across different habitats. For example, 154, 68, 62, 28, and 25 core genera were observed in drip waters, weathered rocks, sediments, bat guanos, and air, which account for 34, 15, 14, 6, and 5% in total 453 known genera, respectively. However, comparative analysis revealed that only 7 core genera were shared by the five habitats. They were *Penicillium, Aspergillus, Mortierella, Acremonium, Trametes, Humicola*, and *Scleroderma*. Furthermore, of the top 20 core genera of each sample, only genera *Mortierella* and *Humicola* were presented in all samples.

### Biodiversity Assessment of Mycobiomes

The alpha diversity indicated that mycobiomes were highly diverse in all the five habitats in Heshang Cave. The dominant phylotypes were fully captured by Illumina HiSeq sequencing as evidenced by a high Good’s coverage (from 99.00 to 99.80%, 99.44% on average, **Table 1**) and plateaued rarefaction curves (Supplementary Figure 2). Community richness and community diversity, as revealed by the Chao1, ACE estimators and Shannon indices, respectively, displayed a similar trend with highest in drip waters, followed by weathered rocks, sediments, bat guanos, and air samples (**Table 1**). Chao1 indices ranged from 207.58 to 1299.84 averaged at 690.45 and ACE index varied from 232.51 to 1349.12 with an average of 702.58. The Shannon’s index ranged from 1.25 to 7.62 (4.52 on average) among 15 samples (**Table 1**). Community richness was significantly different between drip waters and air samples as revealed by Chao1 (Wilcoxon’s test, P < 0.01), ACE (Wilcoxon’s test, *p* < 0.01) and observed species (Wilcoxon’s test, *p* < 0.001). Meanwhile, significant difference between weathered rocks and air samples (Wilcoxon’s test, *p* < 0.01) and difference between drip waters and sediments (Wilcoxon’s test, *p* < 0.05) were also detected as indicated by the above three parameters. Community diversity was significantly different between drip waters and bat guanos (Shannon and Simpson indices, Wilcoxon’s test, *p* < 0.01), while difference between weathered rocks and bat guanos was also observed (Shannon and Simpson indices, Wilcoxon’s test, *p* < 0.05).

**FIGURE 5:**
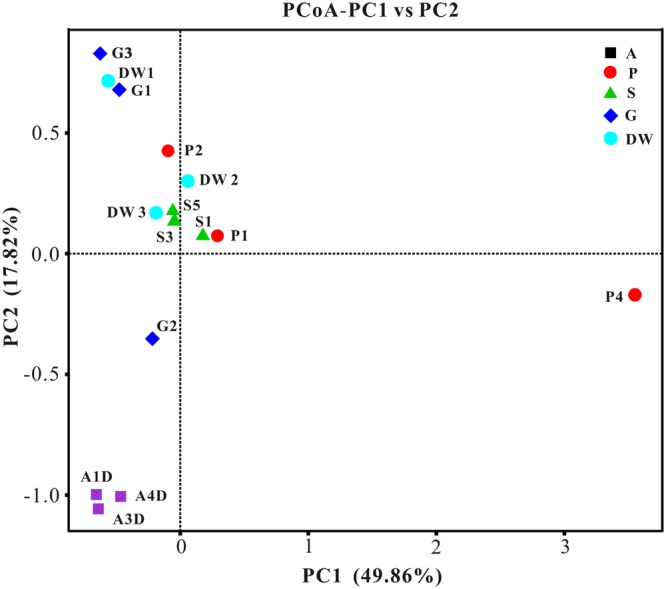
Results of principal coordinate analysis (PCoA) based on weighted-UniFrac distance matrix of mycobiomes in Heshang Cave. Abbreviations are the same as those in **Figure 2**.

**FIGURE 6:**
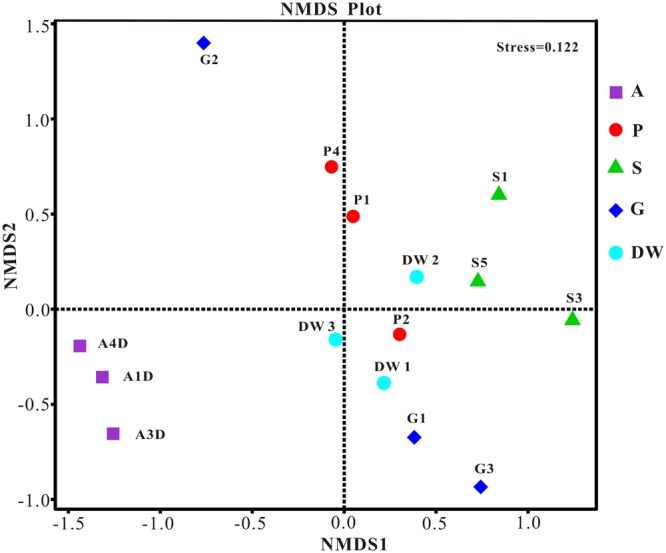
Nonmetric Multidimensional Scaling (NMDS) analysis based on the OTU level of mycobiomes in Heshang Cave. Abbreviations are the same as those in **Figure 2**.

The result of habitat based beta diversity showed no significant difference between two groups (PERMANOVA, *p* > 0.05). Both fungal compositions and beta diversity showed large differences between air and weathered rocks (Supplementary Figure 3, Wilcoxon’s test, *p* < 0.01). We further explored the relationship among samples using PCoA analysis based on the weighted Unifrac distances and the result explained 49.86% of the variation by Axis 1 and 17.82% by Axis 2 (**Figure 5**). Meanwhile, statistical differences in the mycobiome compositions were clearly demonstrated via NMDS analysis at the OTU level and the 15 samples clustered according to habitats (**Figure 6**). Mycobiomes in air samples were distinctive from those of other samples (**Figures 5, 6**). Overlaps of mycobiomes were observed between the other four habitats (**Figure 5**), but mycobiomes can be better separated by multivariate community analysis with NMDS (**Figure 6**). However, the observation of a few exceptional samples via PCoA (G2 and P4) and NMDS (G2 and P2) analysis suggested the heterogeneity of mycobiomes in a specific habitat (**Figures 5, 6**).

**FIGURE 7:**
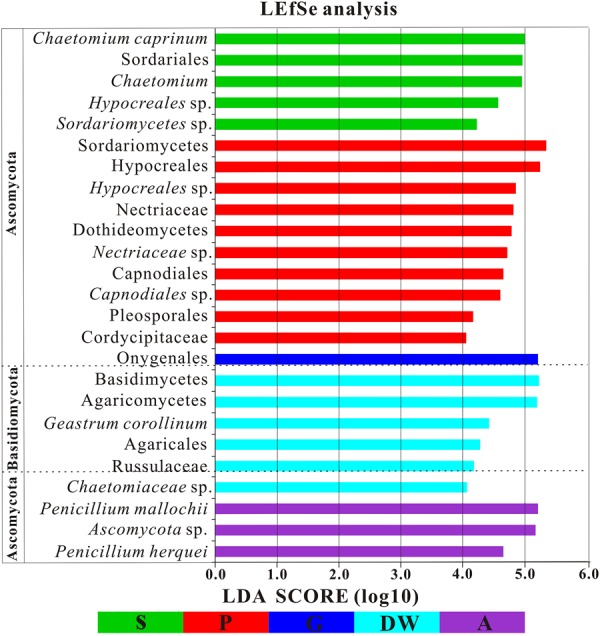
Results of Linear discriminant analysis Effect Size (LEfSe) analysis of mycobiomes in Heshang Cave (LDA Score > 4). Abbreviations are the same as those in **Figure 2**.

The LEfSe analysis was used to distinguish indicator groups of mycobiomes from different habitats using a LDA score threshold of > 4. These statistically and biologically differential clades explain the greatest differences of mycobiomes in Heshang Cave. In total, 25 indicator groups were identified from the five habitats (**Figure 7**). Of those, 7 indicator groups have high LDA scores (LDA > 5, *p* < 0.05) (**Figure 7**), illustrating their specificity and high abundance in a given habitat. Specifically, two species of genus *Penicillium, P. mallochii*, and *P. herquei* (LDA > 4, *p* < 0.05) were significantly enriched in air samples (**Figure 7**). At the order levels, Capnodiales, Pleosporales, and Hypocreales were significantly enriched in weathered rocks, while Sordariales and Agaricales were significantly enriched in sediments and drip waters, respectively (LDA > 4, *p* < 0.05) (**Figure 7**). At family level, Nectriaceae, Cordycipitaceae, and Chaetomiaceae were identified as indicator groups of weathered rocks and drip waters (LDA > 4, *p* < 0.05) (**Figure 7**).

**FIGURE 8:**
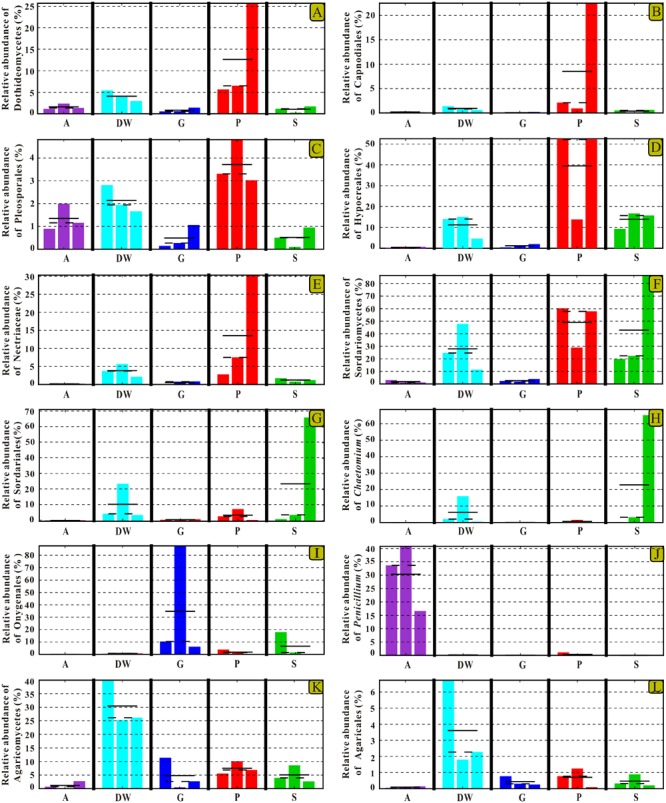
Selected relative abundance histograms of indicator groups detected by LEfSe for mycobiomes in Heshang Cave. Dothideomycetes **(A–C)**, Sordariomycetes **(D–H)**, and Eurotiomycetes **(I,J)** in Ascomycota and Agaricomycetes **(K,L)** in Basidiomycota were displayed. Abbreviations are the same as those in **Figure 2**.

The identified indicator groups mainly affiliated with four classes of Dothideomycetes (**Figures 8A–C**), Sordariomycetes (**Figures 8D–H**), and Eurotiomycetes (**Figures 8I,J**) in Ascomycota and Agaricomycetes (**Figures 8K,L**) in Basidiomycota. Sordariomycetes and Agaricomycetes had a relatively high abundance (> 25%) in drip waters (*t*-test, *p* < 0.05) (**Figures 8F,K**) while Dothideomycetes (*t*-test, *p* < 0.05), Hypocreales (*t*-test, *p* < 0.05), Nectriaceae (Metastats, *p* < 0.05), and *Chaetomium* (Metastats, *p* < 0.05) were lower than 20% (**Figures 8A,D,E,H**). Onygenales was highly abundant in bat guanos (relative abundance > 30%) and less abundant in other four habitats (Metastats, *p* < 0.05; *t*-test, *p* < 0.05) (**Figure 8I**). Sordariomycetes and Hypocreales were highly enriched in weathered rocks (relative abundance > 50%) (*t*-test, *p* < 0.05) (**Figures 8D,F**) than other indicator groups (relative abundance < 10%) (**Figures 8G–L**). However, the abundance of Sordariomycetes and Hypocreales in sediments were relatively low (>10%) in comparison with that in the weathered rocks. Interestingly, air samples showed high abundance of *Penicillium* (relative abundance > 30%) (*t*-test, *p* < 0.05) (**Figure 8**).

## Discussion

### The Dominance of Ascomycota in Heshang Cave

Although caves are considered to be extreme environments to life, our results clearly demonstrated a highly diverse mycobiomes in Heshang Cave than that revealed by culture-dependent methods previously (Man et al., 2015), indicating a high fungal diversity in Heshang Cave. In total 453 genera, 72 orders and 19 classes in 6 phyla of fungi were detected from five habitats in Heshang Cave. Ascomycota (72.25% reads, 42% OTUs) was the most abundant phylum across the five habitats. This result is consistent with previous reports (Vanderwolf et al., 2013), which showed a predominance of Ascomycota (69%) in caves and mines worldwidely. This high throughput sequencing data were also consistent with the result via culture-dependent method conducted in the same cave (Man et al., 2015).

To date, 20 families (13 families of which belong to Ascomycota) of mycota were the most frequently detected in solution caves by culture-dependent methods (Vanderwolf et al., 2013). A total of 65% of them (i.e., Mucoraceae, Mortierellaceae, Fomitopsidaceae, Mycenaceae, Polyporaceae, Trichocomaceae, Nectriaceae, Pleosporaceae, Microascaceae, Hypocreaceae, Davidiellaceae, Arthrodermataceae, and Clavicipitaceae) was also detected in 179 known families in Heshang Cave. Specifically, Trichocomaceae (Ascomycota) was the most abundant family in our study and represented by 14.21% of total reads and 42 OTUs. This result is further supported by Vanderwolf et al. (2013), which shows the dominance of Trichocomaceae in solution caves. The taxa in Trichocomaceae are saprobes with aggressive colonization strategies, capable of surviving in extreme environmental conditions. Some fungi of these taxa, such as *Penicillium* and *Aspergillus*, are not only frequently detected in caves by culture-dependent methods (Man et al., 2015; Ogórek et al., 2016) and NGS technologies aforementioned, but also capable of degrading rocks and solubilizing minerals (Sterflinger, 2000). Therefore, further studies are clearly needed to elucidate the ecology and potential roles of the dominant family Trichocomaceae present in Heshang Cave.

### Fungal Specificity to Habitats

Despite the prevailing harsh and hostile environments, rock surfaces still serve as reservoirs for specialized fungi. Our results confirm the maximum indicator groups in weathered rocks by LEfSe analysis. The result suggested that indicator groups in weathered rocks were more diverse than those in other habitats in Heshang Cave. The class Sordariomycetes (relative abundance of 43%) and the order Hypocreales (relative abundance 34.62%) dominated in weathered rocks. The specificity of Sordariomycetes for rock habitats was strongly supported by the high LDA score (LDA Score > 5, **Figure 7**). Previously, it was reported that rock-inhabiting fungi dwelled on bare rock surfaces such as limestone, sandstone, granite, slate, dolomite, and quartzite and mainly affiliated with two classes of Ascomycota: Dothideomycetes and Eurotiomycetes (Ruibal et al., 2009; Egidi et al., 2014). Our results indicated a dominance of Sordariomycetes (43%) over Dothideomycetes (14.18%) and Eurotiomycetes (2.88%) in weathered rocks (mainly dolomite). Furthermore, 35% of the 20 most common families reported from cave environments (Vanderwolf et al., 2013) were members of Sordariomycetes. Therefore, these in-depth NGS results suggest that Sordariomycetes is a potential main fungal group on weathered dolomite rocks and the diversity of them remains underestimated.

The classes of Agaricomycetes and Tremellomycetes in Basidiomycota were the most abundant and encompassed 6.90 and 2% of sequences derived from weathered rocks, respectively. Tremellomycetes was relatively less abundant taxon in our survey, only represented by 1.46% of total sequences. In contrast, Tremellomycetes represented the most abundant class (40%) in the weathered rocks in Kartchner Caverns (Vaughan et al., 2015). This discrepancy is possibly attributed to the remarkable heterogeneity of fungi at the local scales (Baldrian et al., 2012; Brown and Jumpponen, 2014) and mineralogical difference between Kartchner Caverns (limestone) and Heshang Cave (dolomite) (Vaughan et al., 2015).

Although Dothideomycetes, as the largest class of Ascomycota, is ecologically diverse, it still subordinated to Sordariomycetes and only presented 14.18% of sequences derived from weathered rocks in this study. Of them, the observation of Capnodiales (10.50%) and Pleosporales (3.33%) as main rock inhabiting fungi was consistent with the results in previous studies (Sterflinger, 2000; Ruibal et al., 2009). Strikingly, at the order level, Hypocreales (34.62%) in class Sordariomycetes was dominant in weathered rocks. This result was confirmed by our previous survey via culture-dependent methods, showing a high abundance of Hypocreales (37%) (Man et al., 2015). The present result in combination with our previously cultivation-based data strongly suggested that Hypocreales was a main rock inhabiting fungi.

The more neglected area in pristine solution cave research is the study of cave aerobiology, especially for air fungi. Although this issue is of great interest for cave management at tourist attractions (Docampo et al., 2010; Taylor et al., 2013) and cultural heritage conservation (Martin-Sanchez et al., 2014), only a few studies have been conducted (Ogórek et al., 2013, 2014; Pusz et al., 2015; Ogórek et al., 2016) to date. In this study, mycobiomes in air samples from the entrance to the innermost part of Heshang Cave were revealed, and the beta diversity based on weighted Unifrac analysis showed that air mycobiomes significantly differed from those of four other habitats and clustered as a single group as indicated by PCoA (**Figure 5**) and NMDS (**Figure 6**) analysis. Moreover, the composition and community structure of air samples also showed a great difference with those in the other four habitats investigated. Generally, the air fungal communities in Heshang Cave are characterized by a predominance of *P. mallochii* (mean relative abundance > 30%) and *P. herquei* (mean relative abundance > 9%), which are frequently detected and confirmed to be the indicator groups of airborne fungi by LEfSe analysis in our study (**Figure 8J**). To the best of our knowledge, this is the first report of high abundance of *P. mallochii* and *P. herquei* in the air of pristine solution caves.

Previous studies have already shown that *Penicillium, Aspergillus*, and *Cladosporium* are the most abundant spore type in air of caves (Docampo et al., 2010, 2011). Our work also confirmed the predominance of *Penicillium* (47.87%) and *Aspergillus* (9.10%) in air samples throughout Heshang Cave. Different from the results in Castañar de Ibor Cave (Jurado et al., 2010) and Niedźwiedzia Cave (Ogórek et al., 2014), where the air fungi was dominated by *Cladosporium. Cladosporium* was only detected in a low abundance (0.18%) in our survey. The airborne fungi characterized by high abundance of *Penicillium* and low abundance of *Cladosporium* may possibly relate to the high humidity in Heshang Cave, which favored for spore release of *Penicillium* but was unfavorable for *Cladosporium* (Pasanen et al., 1991). In addition, the diversity of air mycobiomes increased from entrance to the innermost part in Heshang Cave. More specifically, the relative abundance of *Penicillium* and *Aspergillus* is increasing from the entrance (A4D: 14.46%, 0.84%), middle (A3D:17.86%, 1.44%) to the innermost part (A1D: 15.55%, 6.82%). As for the total OTUs of air mycobiomes, fewer OTUs was retrieved in air close to the entrance (A4D: 31.23% of total OTUs in air samples) than that of inner part in Heshang Cave (A3D: 41.29% of total OTUs in air samples). Therefore, the diversity of air mycobiomes decreased from the inner part to the entrance of Heshang Cave. The reasons for this observation are probably attributed to: 1) Microclimate is stable throughout the year (mean 18°C) in the innermost part while conditions are relatively fluctuating at the cave entrance; and 2) The constant saturation of relative humidity (100%) in the interior part promotes the diversity and composition of mycobiomes (Crowther et al., 2014; Peay et al., 2016) and facilitates the release of fungal spores, especially for *Penicillium* (Pasanen et al., 1991).

## Conclusion

Highly diverse mycobiomes were revealed by high-throughput sequencing methods with 453 genera, 72 orders and 19 classes within 6 phyla in five habitats of Heshang Cave. Ascomycota is dominant across the five habitats and account for 72.25% reads and 42% OTUs. Community richness and diversity were highest in drip waters and lowest in air samples. Each habitat has distinctive indicator groups. Among the five habitats investigated, weathered rocks (12%) harbored the maximum distinct OTUs followed by drip waters (9%), and only 6.60% core OTUs were shared by all the 15 samples in this study. We first identified Sordariomycetes (43%) in weathered rocks to be dominant for rock in-habitating fungi. Air samples were dominated by *P. Mallochii* (> 30%) and *P. herquei* (>9%) and the community richness of airborne mycobiome increased from the entrance to the inner part of the cave. Our results will facilitate a better understanding of the highly diverse mycobiomes in Heshang Cave, enabling better predictions of potentially roles of mycobiomes in subterranean ecosystems.

## Author Contributions

BM performed the experiments, analyzed the data, and wrote the manuscript. HW designed the experimental plan, provided the funding, and polished the manuscript. XX and YY assisted with the data analysis. RW, YD, and XC helped with the sampling work.

## Conflict of Interest Statement

The authors declare that the research was conducted in the absence of any commercial or financial relationships that could be construed as a potential conflict of interest.

## Footnotes

1 http://ccb.jhu.edu/software/FLASH/

2 http://qiime.org/index.html

3 https://unite.ut.ee/

4 http://www.drive5.com/usearch/manual/uchime_algo.html

5 http://drive5.com/uparse/

6 http://www.drive5.com/muscle/

7 http://www.ncbi.nlm.nih.gov/bioproject/352141
