# Supplementary material for: Diversity of Fungal Communities in Heshang Cave of Central China Revealed by Mycobiome-Sequencing
Source: Front Microbiol. 2018 Jul 16;9:1400. doi: 10.3389/fmicb.2018.01400 (PMC6054936; doi:10.3389/fmicb.2018.01400)
Supplement: Supplementary file 1 [file Table_1.pdf]

Supplementary Table 1

Diversity of fungal communities in Heshang Cave of central China revealed by mycobiome-sequencing

Baiying Man[1,2], Hongmei Wang[1,3 *], Yuan Yun[1], Xing Xiang[1], Ruicheng Wang[1], Yong Duan[1] and Xiaoyu Cheng[1]

[1] State Key Laboratory of Biogeology and Environmental Geology, China University of Geosciences, Wuhan, P R China

[2] College of life science, Shangrao Normal University, Shangrao, P R China

[3] Laboratory of Basin Hydrology and Wetland Eco-restoration, China University of Geosciences, Wuhan, P R China

*** Corresponding author. E-mail:** hmwang@cug.edu.cn or wanghmei04@163.com

Telephone：86-13419513876； +86-27-67883158；Fax number：+86-27-87436235

Supplementary Table 1 Primer and barcode information for Illumina HiSeq sequencing of mycobiomes in Heshang Cave, central China.

Sample	Barcode-F	Barcode-F Sequence	Linker Primer Sequence ITS5-1737F	Barcode-R	Barcode-R Sequence	Linker Primer Sequence ITS2-2043R
A1D	barcode5	ACAGTG	GGAAGTAAAAGTCGTAACAAGG	barcode22	CGTACG	GCTGCGTTCTTCATCGATGC
A3D	barcode6	GCCAAT	GGAAGTAAAAGTCGTAACAAGG	barcode11	GGCTAC	GCTGCGTTCTTCATCGATGC
A4D	barcode6	GCCAAT	GGAAGTAAAAGTCGTAACAAGG	barcode12	CTTGTA	GCTGCGTTCTTCATCGATGC
P1	barcode1	ATCACG	GGAAGTAAAAGTCGTAACAAGG	barcode2	CGATGT	GCTGCGTTCTTCATCGATGC
P2	barcode1	ATCACG	GGAAGTAAAAGTCGTAACAAGG	barcode13	AGTCAA	GCTGCGTTCTTCATCGATGC
P4	barcode5	ACAGTG	GGAAGTAAAAGTCGTAACAAGG	barcode14	AGTTCC	GCTGCGTTCTTCATCGATGC
S1	barcode1	ATCACG	GGAAGTAAAAGTCGTAACAAGG	barcode5	ACAGTG	GCTGCGTTCTTCATCGATGC
S3	barcode1	ATCACG	GGAAGTAAAAGTCGTAACAAGG	barcode6	GCCAAT	GCTGCGTTCTTCATCGATGC
S5	barcode1	ATCACG	GGAAGTAAAAGTCGTAACAAGG	barcode7	CAGATC	GCTGCGTTCTTCATCGATGC
G1	barcode1	ATCACG	GGAAGTAAAAGTCGTAACAAGG	barcode18	GTCCGC	GCTGCGTTCTTCATCGATGC
G2	barcode5	ACAGTG	GGAAGTAAAAGTCGTAACAAGG	barcode20	GTGGCC	GCTGCGTTCTTCATCGATGC
G3	barcode1	ATCACG	GGAAGTAAAAGTCGTAACAAGG	barcode14	AGTTCC	GCTGCGTTCTTCATCGATGC
DW1	barcode1	ATCACG	GGAAGTAAAAGTCGTAACAAGG	barcode15	ATGTCA	GCTGCGTTCTTCATCGATGC
DW2	barcode1	ATCACG	GGAAGTAAAAGTCGTAACAAGG	barcode16	CCGTCC	GCTGCGTTCTTCATCGATGC
DW3	barcode1	ATCACG	GGAAGTAAAAGTCGTAACAAGG	barcode17	GTAGAG	GCTGCGTTCTTCATCGATGC
