# Supplementary material for: Diversity of Fungal Communities in Heshang Cave of Central China Revealed by Mycobiome-Sequencing
Source: Front Microbiol. 2018 Jul 16;9:1400. doi: 10.3389/fmicb.2018.01400 (PMC6054936; doi:10.3389/fmicb.2018.01400)
Supplement: Supplementary file 2 [file Table_2.pdf]

Supplementary Table 2

Diversity of fungal communities in Heshang Cave of central China revealed by mycobiome-sequencing

Baiying Man[1,2], Hongmei Wang[1,3 *], Yuan Yun[1], Xing Xiang[1], Ruicheng Wang[1], Yong Duan[1] and Xiaoyu Cheng[1]

[1] State Key Laboratory of Biogeology and Environmental Geology, China University of Geosciences, Wuhan, P R China

[2] College of life science, Shangrao Normal University, Shangrao, P R China

[3] Laboratory of Basin Hydrology and Wetland Eco-restoration, China University of Geosciences, Wuhan, P R China

*** Corresponding author. E-mail:** hmwang@cug.edu.cn or wanghmei04@163.com

Telephone：86-13419513876； +86-27-67883158；Fax number：+86-27-87436235

Supplementary Table 2 Physicochemical parameters of samples in Heshang Cave.

Sample	pH	Ca^{2+} (mM)	Mg^{2+} (mM)	Cl^{-} (mM)	NO_3^{-} (mM)	SO_4^{2-} (mM)
S1	8.08	1.28	2.52	1.05	4.81	0.18
S3	8.15	3.53	7.95	2.81	4.04	2.41
S5	8.05	1.17	0.73	0.33	2.01	0.37
P1	7.74	5.60	24.25	1.65	6.38	19.77
P2	7.84	2.34	2.65	1.79	4.60	2.04
P4	7.80	4.97	2.65	0.81	8.33	9.59
G1	6.60.	10.65	12.37	1.81	11.90	3.02
G2	6.39	8.35	8.46	2.09	13.45	2.41
G3	6.63	11.90	13.08	4.11	38.22	4.18
DW1	7.86	0.89	1.50	0.08	0.14	0.33
DW2	7.96	0.87	1.52	0.06	0.22	0.28
DW3	7.76	0.68	1.85	0.05	0.20	0.33
