# Supplementary material for: Diversity of Fungal Communities in Heshang Cave of Central China Revealed by Mycobiome-Sequencing
Source: Front Microbiol. 2018 Jul 16;9:1400. doi: 10.3389/fmicb.2018.01400 (PMC6054936; doi:10.3389/fmicb.2018.01400)
Supplement: Supplementary file 4 [file Image_1.pdf]

*Supplementary Figures*

# Diversity of fungal communities in Heshang Cave of central China revealed by mycobiome-sequencing

Baiying Man[1,2], Hongmei Wang[1,3 *], Yuan Yun[1], Xing Xiang[1], Ruicheng Wang[1], Yong Duan[1] and Xiaoyu Cheng[1]

[1] State Key Laboratory of Biogeology and Environmental Geology, China University of Geosciences, Wuhan, P R China

[2] College of life science, Shangrao Normal University, Shangrao, P R China

[3] Laboratory of Basin Hydrology and Wetland Eco-restoration, China University of Geosciences, Wuhan, P R China

*** Corresponding author. E-mail:** hmwang@cug.edu.cn or wanghmei04@163.com

Telephone：86-13419513876； +86-27-67883158；Fax number：+86-27-87436235

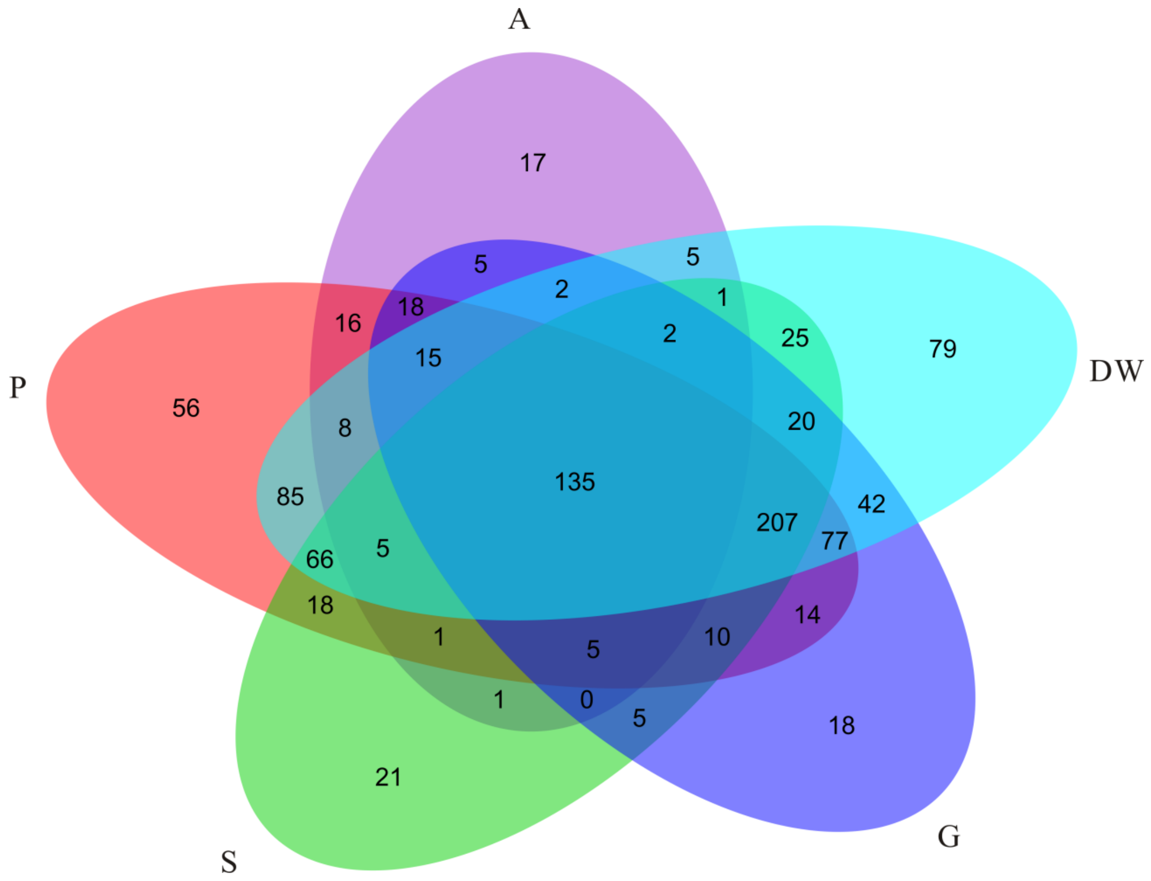


**Supplementary Figure 1**. Venn diagram displayed the number of OTUs at the genus level for mycobiomes of five habitats in Heshang Cave. Abbreviations are the same as those in Figure 2.

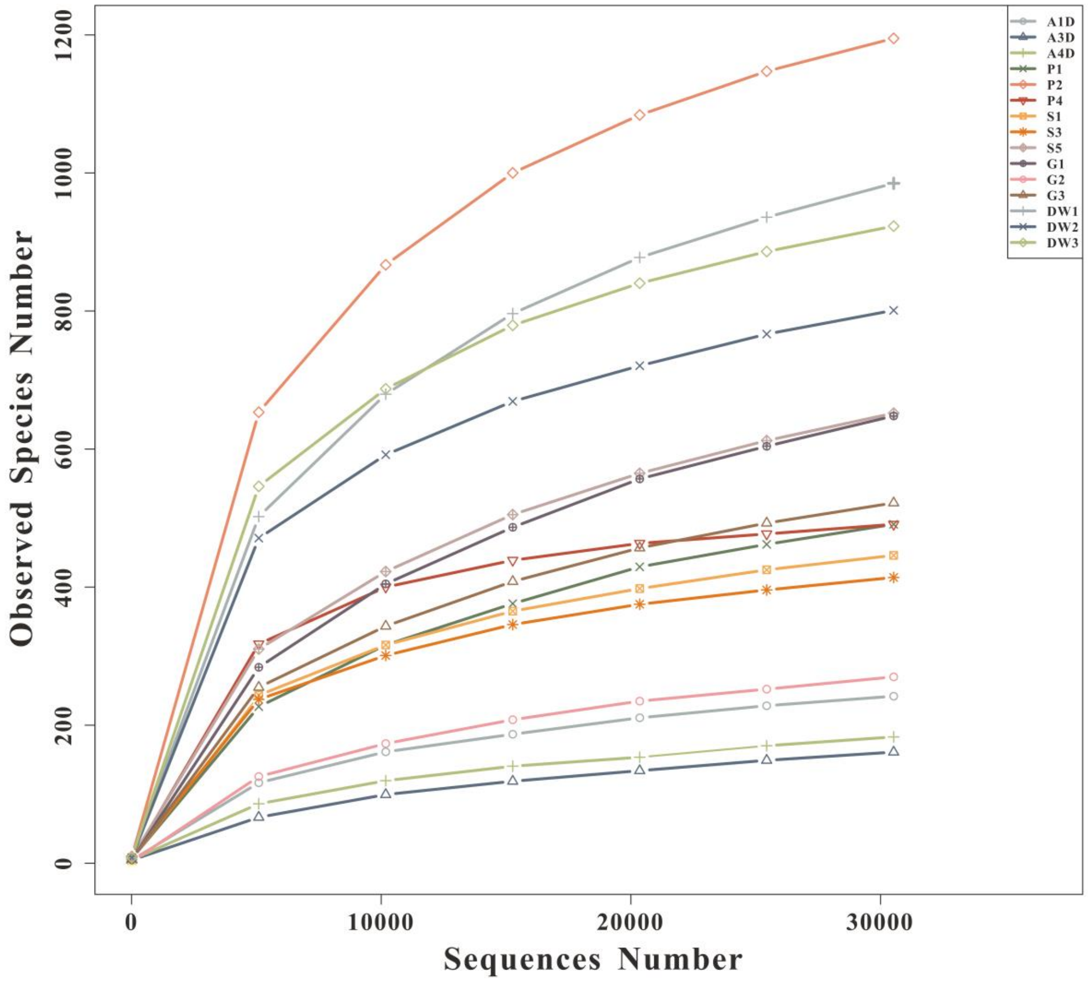


**Supplementary Figure 2**. Rarefaction curves of mycobiomes based on OTUs of the fifteen samples in Heshang Cave. Abbreviations are the same as those in Figure 2.

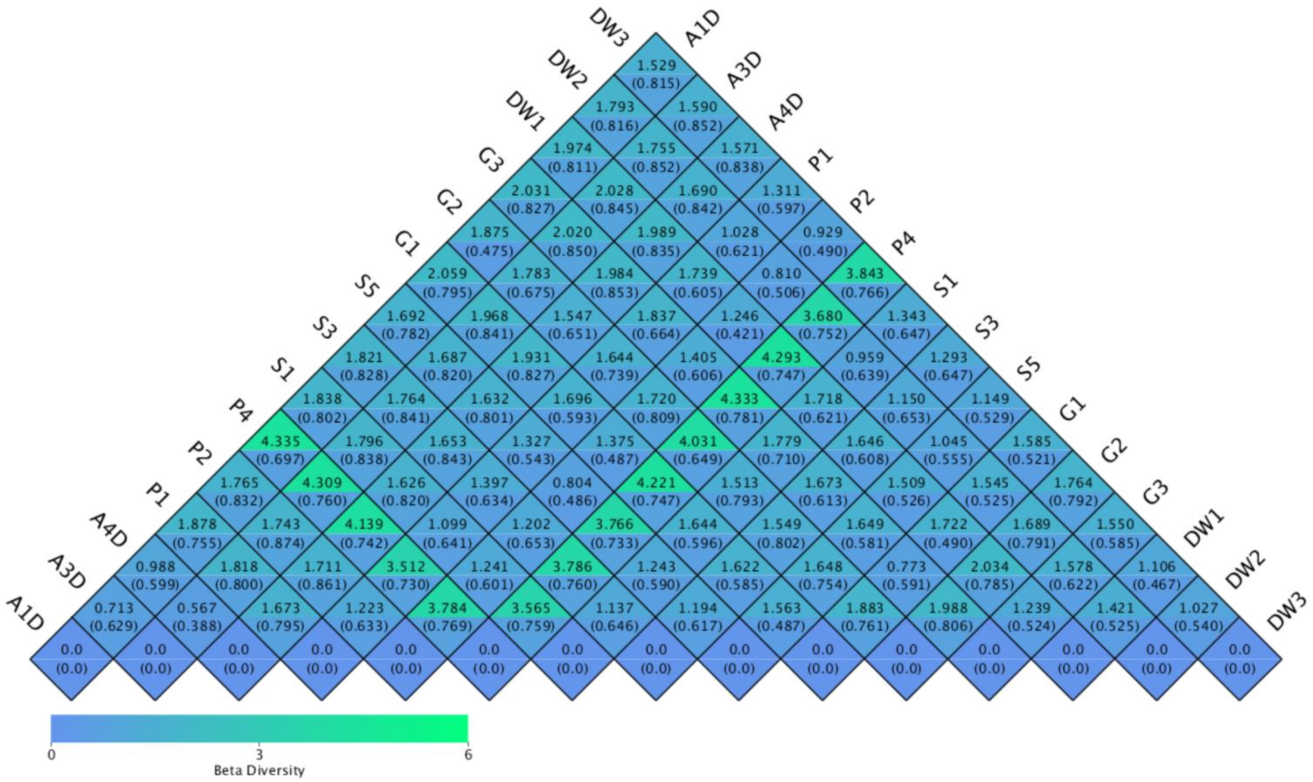


**Supplementary Figure 3**. The heatmap of beta diversity index. Comparisons of discrepancy coefficient between samples via weighted unifrac (uper) and unweighted unifrac (bottom) distances were given in the same diamond. The smaller of the discrepancy coefficient, the smaller of differences for the diversity of species. Abbreviations are the same as those in Figure 2.
